# Analysis of Cryptococcal Extracellular Vesicles: Experimental Approaches for Studying Their Diversity Among Multiple Isolates, Kinetics of Production, Methods of Separation, and Detection in Cultures of Titan Cells

**DOI:** 10.1128/spectrum.00125-21

**Published:** 2021-08-04

**Authors:** Flavia C. G. Reis, Bianca Gimenez, Luísa J. Jozefowicz, Rafael F. Castelli, Sharon T. Martins, Lysangela R. Alves, Haroldo C. de Oliveira, Marcio L. Rodrigues

**Affiliations:** a Instituto Carlos Chagas, Fundação Oswaldo Cruz (Fiocruz), Curitiba, Brazil; b Centro de Desenvolvimento Tecnológico em Saúde (CDTS), Fiocruz, Rio de Janeiro, Brazil; c Instituto de Microbiologia Paulo de Góes (IMPG), Universidade Federal do Rio de Janeiro, Rio de Janeiro, Brazil; Institut Pasteur

**Keywords:** *Cryptococcus*, extracellular vesicles, pathogenesis

## Abstract

Extracellular vesicles (EVs) produced by members of the Cryptococcus genus are associated with fundamental processes of fungal physiology and virulence. However, several questions about the properties of cryptococcal EVs remain unanswered, mostly because of technical limitations. We recently described a fast and efficient protocol of high-yield EV isolation from solid medium. In this study, we aimed at using the solid medium protocol to address some of the open questions about EVs, including the kinetics of EV production, the diversity of EVs produced by multiple isolates under different culture conditions, the separation of vesicles in a density gradient followed by the recovery of functional EVs, the direct detection of EVs in culture supernatants, and the production of vesicles in solid cultures of Titan cells. Our results indicate that the production of EVs is directly impacted by the culture medium and time of growth, resulting in variable detection of EVs per cell and a peak of EV detection at 24 h of growth. Nanoparticle tracking analysis (NTA) of EV samples revealed that multiple isolates produce vesicles with variable properties, including particles of diverging dimensions. EVs were produced in the solid medium in amounts that were separated on a centrifugation density gradient, resulting in the recovery of functional EVs containing the major cryptococcal capsular antigen. We also optimized the solid medium protocol for induction of the formation of Titan cells, and analyzed the production of EVs by NTA and transmission electron microscopy. This analysis confirmed that EVs were isolated from solid cultures of cryptococcal enlarged cells. With these approaches, we expect to implement simple methods that will facilitate the analysis of EVs produced by fungal cells.

**IMPORTANCE** Fungal extracellular vesicles (EVs) are considered to be important players in the biology of fungal pathogens. However, the limitations in the methodological approaches to studying fungal EVs impair the expansion of knowledge in this field. In the present study, we used the Cryptococcus genus as a model for the study of EVs. We explored the simplification of protocols for EV analysis, which helped us to address some important, but still unanswered, questions about fungal EVs.

## INTRODUCTION

Fungal extracellular vesicles (EVs) were first described in Cryptococcus neoformans in 2007 (1). Since then, the phenomenon of EV production has been studied in detail in C. neoformans and Cryptococcus gattii (2–7) and has been expanded to other species, including Histoplasma capsulatum (8), Saccharomyces cerevisiae (9), Malassezia sympodialis (10), Paracoccidioides brasiliensis (11), Candida albicans (12), Candida glabrata (13), Candida tropicalis, Candida parapsilosis (8, 13), Candida auris (14), Trichophyton interdigitale (15), Sporothrix brasiliensis (16), Trichoderma reesei (17), Aspergillus fumigatus (18), Fusarium oxysporum (19), Zymoseptoria tritici (20), Alternaria infectoria (21), Penicillium digitatum (22), Pichia fermentans (23), and Talaromyces marneffei (24). In this group of 20 species, we estimate that EVs were detected in samples produced by more than 50 fungal isolates.

Fungal EVs participate in the activation of protective immune responses (25), cell-to-cell communication (4), prion transmission (26), antifungal resistance (27), and biofilm formation (23). However, several questions remain unanswered. For instance, the mechanisms of biogenesis of fungal EVs are only superficially known (28), which impairs the development of pharmacological and genetic tools to control EV formation. This limitation likely has an impact on the understanding of how EVs affect fungal pathogenesis. Fungal EVs are promising vaccine candidates (25), but it is still unknown if EV production can be scaled up *in vitro*. In fact, the best conditions for efficient EV production by fungal cells are not known. The literature still lacks information on whether fungal EVs are produced during infection, which negatively affects the design of physiological and/or pathogenic models investigating the pathogenic roles of these structures. Finally, most of the studies on fungal EVs were based on the analysis of EVs produced by yeast forms of fungi. Although EVs were detected in biofilms (23, 27), spores (18), and filamentous forms of fungi (18, 21, 22), it is still unclear if other morphological stages produce EVs. This is likely an important experimental issue to be addressed, since morphological transition is linked to virulence in several fungal pathogens (29). In Cryptococcus, cellular differentiation into enlarged Titan cells is an important virulence determinant (30).

Many of the literature gaps illustrated above derive from the lack of appropriate experimental approaches to analyze fungal EVs. For instance, the understanding of how mammalian EVs are formed greatly benefited from the compositional analysis of EVs separated in density gradients according to their biophysical properties, which lately reflect their sites of biogenesis (31). In fungi, gradient separation was superficially explored in a few studies (1, 32). Our empirical laboratory experience indicates that fungal EV gradients are not generally functional because of the reduced amounts of vesicles typically obtained from cryptococcal cultures. This kind of limitation, for instance, impairs our understanding of how fungal EVs are formed, since the compositional analyses performed so far included a mixture of EVs with similar sedimentation properties, but of different cellular origins (33). For similar reasons, the small molecule composition of cryptococcal EVs has not been feasibly assessed until very recently (7).

EVs are usually recovered from liquid cultures of prokaryotic or eukaryotic cells, followed by sedimentation of vesicle particles by ultracentrifugation. In models where EV production is not abundant, vesicle isolation can require large volumes of culture supernatants. We recently described a facilitated protocol for the isolation of fungal EVs (2). This protocol was based on the recovery of EVs from solid cultures. One important advantage of this approach is the control of the volumes that will be centrifuged. For instance, instead of processing 0.5 to 2 liters of cultures before ultracentrifuging the EV suspension, solid cultures on agar plates can be combined in 15- to 30-ml suspensions, which greatly improves the efficiency of the protocol. This protocol was successfully applied for the isolation of EVs from C. neoformans, Cryptococcus deuterogattii, S. cerevisiae, H. capsulatum, C. albicans, and *P. digitatum* (2, 22). The method was also successfully applied for the preparation of cryptococcal EVs for vaccinal and compositional tests (34).

In this study, we used the solid medium protocol to address remaining questions about fungal EVs. Using Cryptococcus as a model, we analyzed the diversity in the diameter of EVs produced by multiple isolates, in addition to different culture conditions. We also separated cryptococcal EVs on a centrifugation density gradient and recovered functional fractions containing EVs. To address whether EVs could be isolated from cultures of Titan cells, we optimized the formation of enlarged cells using a solid version of the medium typically applied for induction of cryptococcal enlargement, for further isolation of vesicles. Our results suggest that the methods of isolation of fungal EVs from solid cultures can be used to tackle several experimental gaps in this emerging field.

## RESULTS

### EVs produced by different isolates of Cryptococcus are diverse.

**FIG 1:**
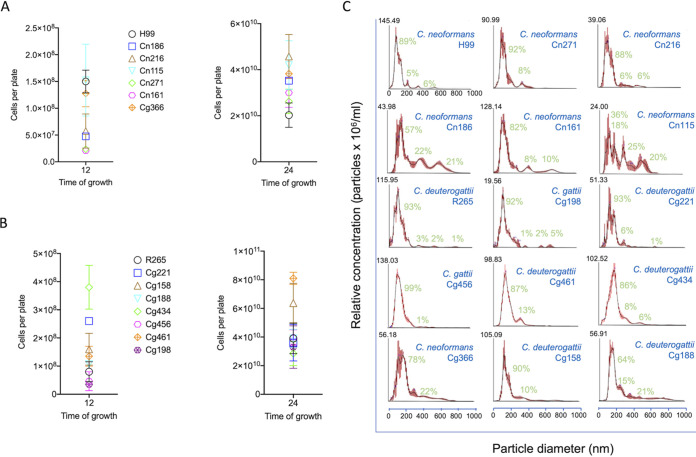
Diversity of EVs in the Cryptococcus genus. (A and B) Growth rates of each of the isolates (see Materials and Methods for strain identification) were determined after cultivation for 12 (A) or 24 (B) h. Although the growth rates were not identical, they were statistically similar (*P* > 0.05, ANOVA). (C) NTA of EVs produced by the multiple isolates of Cryptococcus. EVs were isolated from each isolate after growth in solid medium and observed by NTA. Although many isolates produced samples with similar properties, some of the C. neoformans strains produced EV populations enriched in larger diameter ranges. Peak areas in each NTA histogram were determined using the Image J software. In each sample, the sum of the areas of all peaks corresponded to 100%, and the percentage values described in green correspond to the relative area of each peak in individual samples. These results illustrate one out of three experiments producing similar results.

Most of the studies on EVs produced by Cryptococcus and other genera were based on a few standard isolates. Therefore, the possibility that the properties of EVs vary in larger sets of isolates cannot be ruled out. Since EV properties directly impact their functions (31), we isolated EVs from a collection of 15 C. neoformans and C. gattii*/*C. deuterogatii isolates (C. neoformans, *n* = 6; *C. deuterogattii*, *n* = 7; C. gattii, *n* = 2). Considering that EV production is evidently impacted by cellular physiology and metabolism in general, we first examined the growth rates of each isolate under the conditions used for EV isolation (Fig. 1A and B). After 12 h of growth, the isolates manifested variable growth abilities. However, after 24 h, there were no significant differences in the proliferation rates of all isolates. The results observed for C. neoformans and C. gattii*/C. deuterogatii* isolates were similar. We then submitted the EVs produced by each member of this isolate collection to NTA. In all samples, the typical peak of EV detection in the 50 to 300 nm range was observed, including in samples produced by the standard strains H99 of C. neoformans and R265 of *C. deuterogattii* (Fig. 1C). Minor peaks at increased size ranges were also observed in these strains and several of the other isolates. Indeed, the general pattern observed for the EVs produced by these two standard strains was observed in most of the isolates. However, some of the isolates of C. neoformans produced EV samples with a clear enrichment in regions of increased dimensions, with hefty peaks at the 200 to 300, 300 to 400, 400 to 500, and even 600 to 800 nm ranges. Although these properties were not observed in the non-*neoformans* isolates, the large peaks did not seem to represent a typical pattern of the C. neoformans isolates, since these peaks were very minor in three of these strains. The functional effect of this observation is not known, but it is clear that different strains of Cryptococcus have the ability of producing EVs with highly variable dimensions under the same conditions. These results are summarized in Table 1.

**TABLE 1:** Quantitative determination^*a*^ of the major peaks found in EV samples of different cryptococcal isolates

Isolate	Species	Major peak (% relative area)	Secondary peak(s) (% relative areas)
H99	C. neoformans	0–200 nm (89)	200–300 nm (5) 350–450 nm (6)
Cn186	C. neoformans	0–300 nm (57)	350–500 nm (22) 500–800 nm (6)
Cn271	C. neoformans	0–300 nm (92)	300–500 nm (8)
Cn161	C. neoformans	0–300 nm (82)	400–500 nm (8) 600–900 nm (10)
Cn216	C. neoformans	0–300 nm (88)	300–450 nm (6) 450–600 nm (6)
Cn115	C. neoformans	0–200 nm (36)	200–300 nm (18) 300–400 nm (25) 400–700 nm (20)
Cg366	C. neoformans	0–300 nm (78)	300–800 nm (22)
R265	*C. deuterogattii*	0–300 nm (93)	300–400 nm (3) 500–600 nm (2) 800–900 nm (1)
Cg456	C. gattii	0–300 nm (92)	300–600 nm (1)
Cg198	*C. deuterogattii*	0–350 nm (89)	350–450 nm (1) 550–650 nm (2) 650–750 (5)
Cg461	*C. deuterogattii*	0–200 nm (87)	300–400 nm (13)
Cg158	*C. deuterogattii*	0–200 nm (90)	200–400 nm (10)
Cg221	*C. deuterogattii*	0–300 nm (93)	300–500 nm (6) 700–800 nm (1)
Cg434	*C. deuterogattii*	0–300 nm (86)	300–400 nm (8) 400–600 nm (6)
Cg188	*C. deuterogattii*	0–200 nm (64)	200–300 nm (15) 300–900 nm (21)

a Quantitative determination was performed with the NTA histograms illustrated in Fig. 1, using the Image J software, as described in the Materials and Methods section.

### Separation of EVs on a centrifugation density gradient.

**FIG 2:**
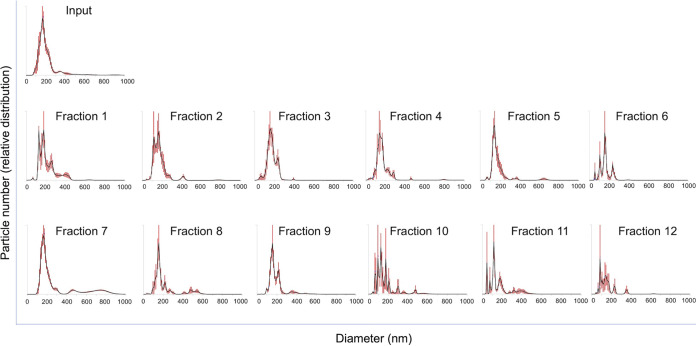
Separation of *C. deuterogattii* EVs on a density gradient. Fungal cells (R265 strain) were cultivated on solid medium for EV isolation. The EVs were applied to the top of an iodixanol gradient and ultracentrifuged for 18 h. The highest fraction numbers represent the densest fractions. Each of 12 fractions was submitted to NTA. Fraction 7 was the one containing the largest population of EVs of higher diameters. This experiment was repeated three times producing similar results.

The results illustrated in Fig. 1 and in several reports in the literature (reviewed in reference 35) suggest that ultracentrifugation pellets of EVs contain mixed populations of vesicles. Early attempts by our group to separate cryptococcal EVs from liquid cultures through gradient centrifugation resulted in the detection of fractions with variable concentrations of GXM (32), the major capsular component of the cryptococcal capsule, which is exported within EVs (1). However, the EV properties in each fraction were not analyzed in these early studies due to the difficulty of recovering detectable EVs in these fractions. In this context, we prepared EVs from solid cultures for gradient separation, aiming at analyzing the properties of each fraction by NTA. In this analysis, we used strain R265 of *C. deuterogattii* grown in yeast extract-peptone-dextrose (YPD) and iodixanol as the density-making reagent. We recovered each fraction from the gradient and submitted them to NTA for diameter determination and particle quantification. Most fractions produced NTA profiles that resembled the typical distribution of unseparated cryptococcal EVs produced in solid YPD by the R265 strain (Fig. 2). Fractions 7 and 8, however, had peaks in the 400 to 800 nm range that were not seen in other samples. Particle quantification (further detailed in Fig. 4A) indicated that fractions 2, 4 to 6, and 10 to 12 gave NTA signals close to the background levels, imposing doubts about their authenticity as vesicular fractions.

**FIG 3:**
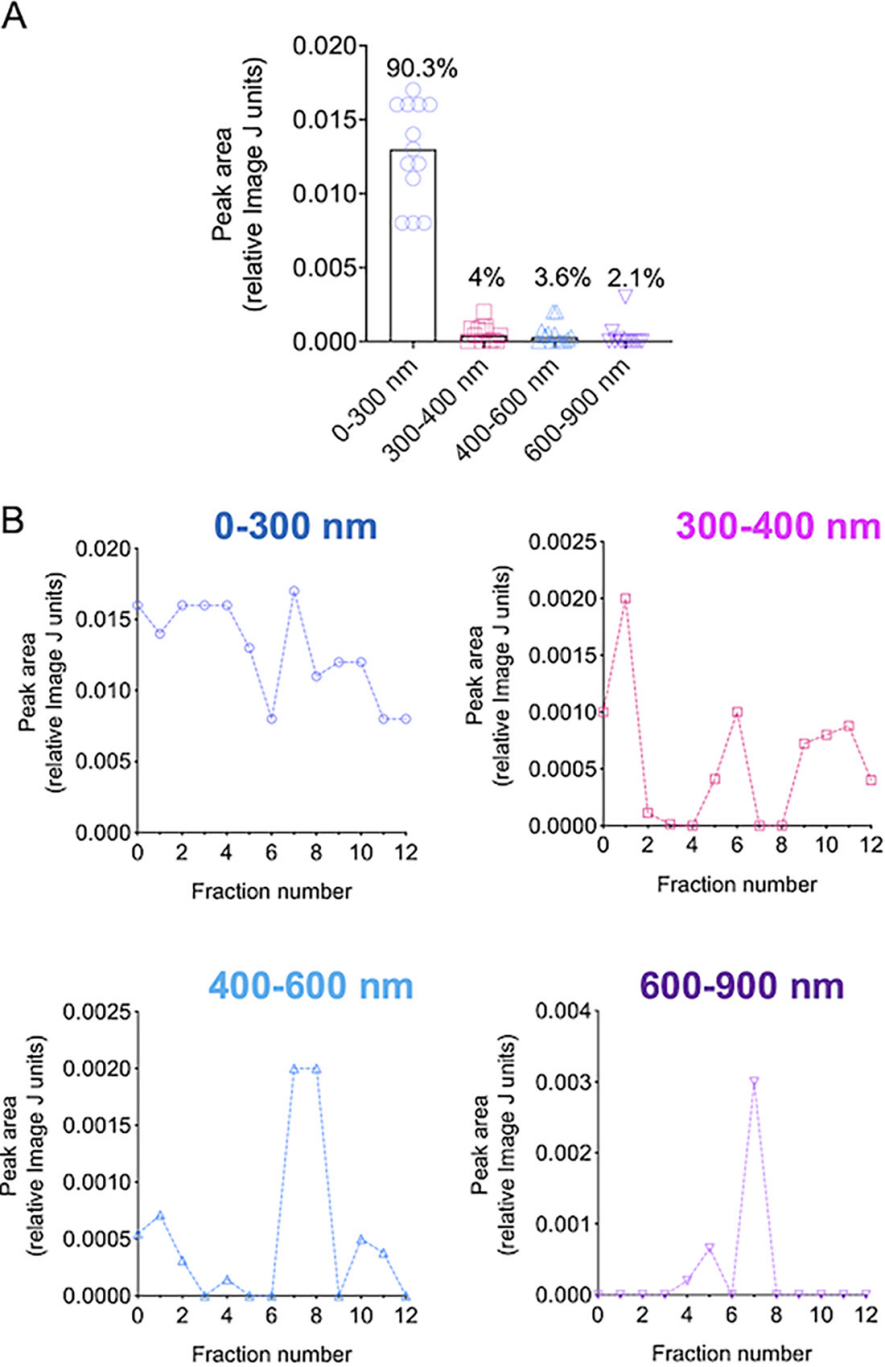
Quantification of the different types of EVs isolated from solid medium using the Image J software. (A) Quantification of EVs in the ranges of 300 to 400 nm (4%), 400 to 600 nm (3.6%), and 600 to 900 nm combining all samples, with a clear predomination of the 0 to 300 diameter range. (B) The analysis of each diameter range is demonstrated as follows: top left, 0 to 300 nm diameter range; top right, 300 to 400 nm range; bottom left, 400 to 600 nm range; and bottom right, 600 to 900 nm size range. Larger diameters predominated in fractions 7 and 8. The quantification results illustrated here derive from the histograms presented in Fig. 2.

Because of the observation of NTA peaks corresponding to EV samples of variable diameters, we determined the area of each peak using the Image J software, aiming at providing quantitative information on the EV populations produced by Cryptococcus. Considering all samples obtained after gradient separation, approximately 90% of the cryptococcal EVs were in the 0 to 300 nm diameter range. Other diameter ranges included 300 to 400 nm (4%), 400 to 600 nm (3.6%), and 600 to 900 nm (2.1%) (Fig. 3A). The analysis of each diameter range in individual gradient samples revealed that the 0 to 300 nm diameter range was not highly variable in the EV samples (Fig. 3B). The 300 to 400 nm range was clearly more evident in fractions 1, 5 to 6, and 9 to 12. Fractions 7 to 8 were evidently enriched in the 400 to 600 nm range (Fig. 3C), and the 600 to 900 nm size range was only detected in fraction 7.

**FIG 4:**
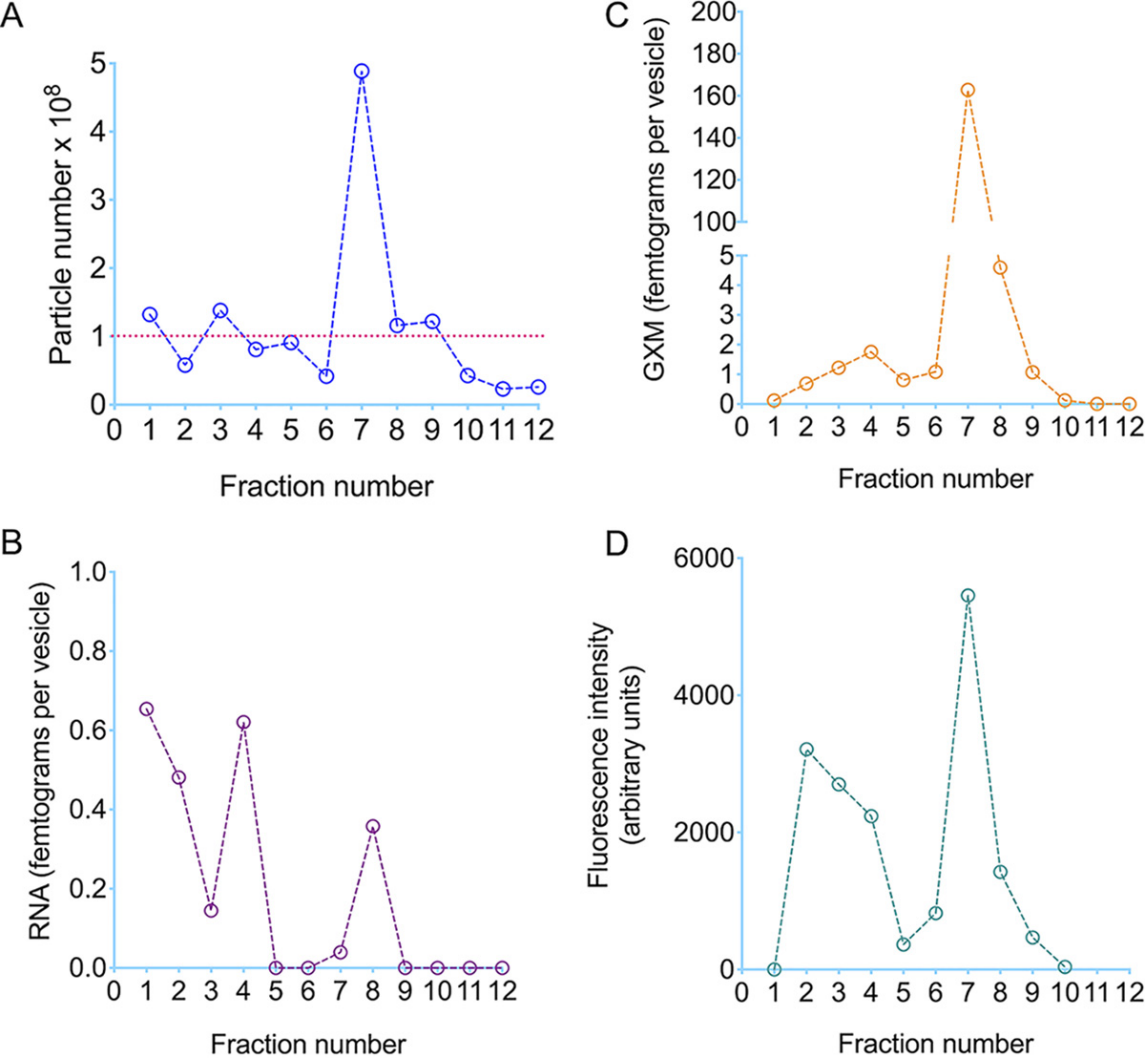
Analysis of particle number (A), RNA detection (B), GXM quantification (C), and the use of gradient fractions by an acapsular mutant of C. neoformans to make a GXM coat (D). (A) The highest particle number was detected in fraction 7. Particle numbers lower that 1 × 10^8^/ml (dashed line) were considered to be at the background levels of EV detection. (B) RNA concentration was normalized in each fraction to the number of particles. The highest concentration of RNA above the background level of EV detection was found in fraction 8. (C) GXM was highly concentrated in fraction 7, even after normalization of the GXM content to the number of particles. (D) Incorporation of GXM from EV fractions by an acapsular mutant of C. neoformans followed by indirect quantification of GXM coating by flow cytometry. Once again, fraction 7 gave the most positive signals of GXM detection. These results illustrate one out of two experiments producing similar results.

All fractions had their EV concentrations determined (Fig. 4A) and were tested for the presence of RNA (Fig. 4B) and GXM (Fig. 4C), two EV components rapidly detected by sensitive tests. Fractions 1, 4, and 8 contained the highest concentrations of RNA, suggesting that this EV component is distributed into vesicles of different cellular nature. GXM detection, however, was clearly more pronounced in fraction 7, the one containing larger vesicles. These results confirmed that ultracentrifugation pellets of cryptococcal EVs contain mixed populations, and suggested that larger EVs are associated with the export of GXM.

We then asked whether the EV fractions separated on the centrifugation gradient could be recovered in their functional forms. To address this question, we used a model based on the well-known reported ability of acapsular C. neoformans cells to use EVs as a source of GXM for surface coating. Acapsular cells were incubated with each fraction and then assessed by flow cytometry after staining with a monoclonal antibody (MAb) to GXM (Fig. 4D). EV gradient fractions 2 to 4 and 7 to 8 were efficient donors of GXM, as concluded from the strong staining of the acapsular mutant cells with the MAb to GXM. Undoubtedly, fraction 7 was the most efficiently used by acapsular cells to form a GXM coat, which was consistent with the highest concentration of GXM.

### Direct detection of EV particles in supernatants suggests an association between larger vesicles and capsule enlargement.

**FIG 5:**
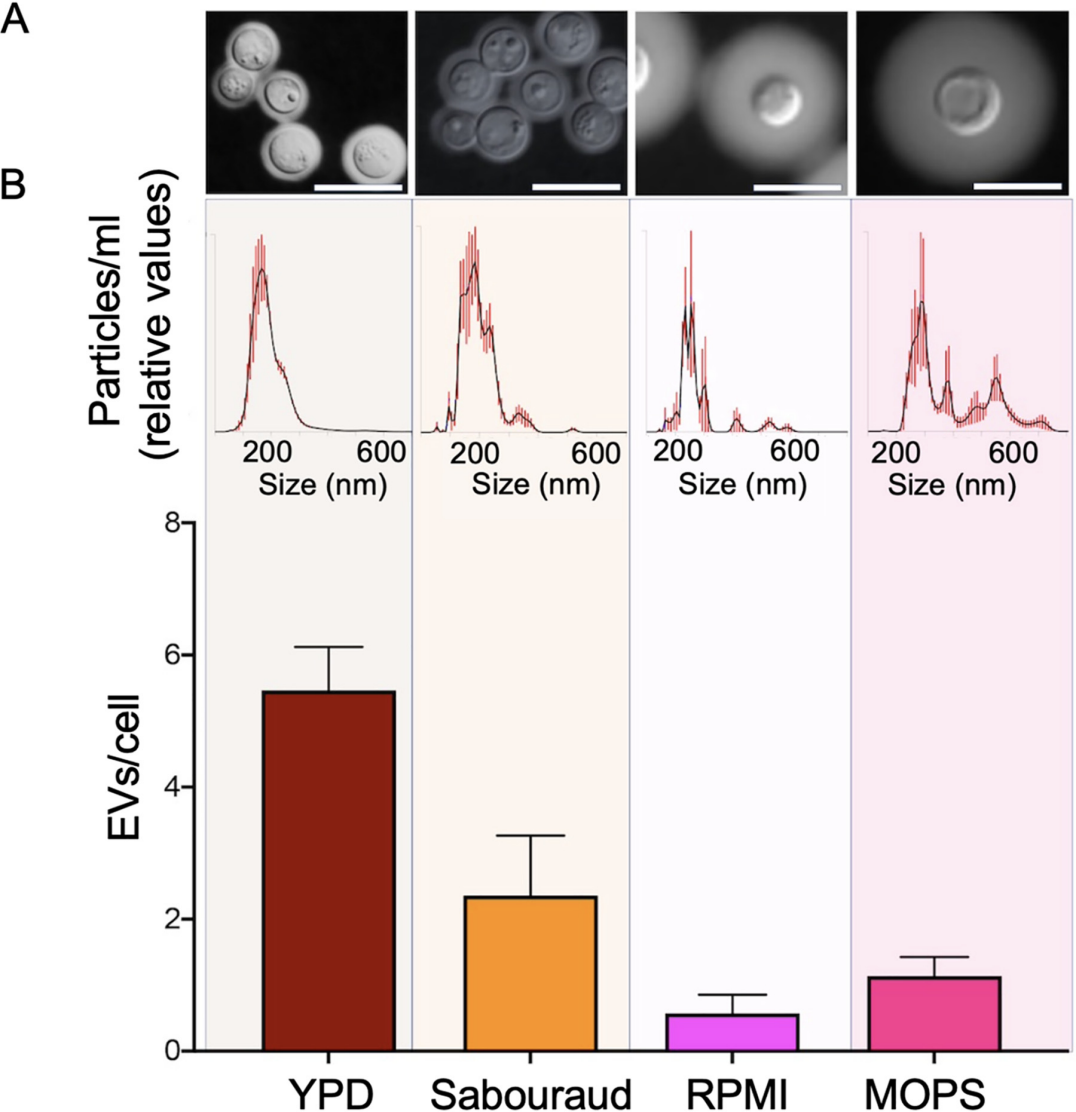
Capsule induction and detection of EVs in culture supernatants. (A) Capsule counterstaining of *C. deuterogattii* (R265) in different culture media. Scale bar, 10 μm. (B) Direct detection of EVs in the supernatants obtained from the culture conditions in A by NTA. In B, the upper panel shows the general NTA aspects of cryptococcal EVs. The lower panel represents the number of EVs detected to the number of cells in the original culture. These results illustrate one out of three experiments producing similar results.

The gradient separation results were suggestive of an association of larger EV fractions with the presence of GXM. To address this hypothesis, we included another previously untested EV experimental model in our analysis: the direct detection of EVs in culture supernatants. We incubated the strain R265 of *C. deuterogattii* under previously established conditions of repression (YPD and Sabouraud) or stimulation (RPMI or 10% Sabouraud in 50 mM MOPS [morpholinepropanesulfonic acid], pH 7.3) of capsule formation at 37°C (36, 37). We collected the cells by centrifugation and counterstained them with India ink for capsule visualization. The supernatants were filtered through 0.8-μm membranes to remove cell debris and then directly submitted to NTA without any further processing. Fungal cells obtained from YPD and Sabouraud had very small capsules, while a moderate induction was observed in RPMI. In MOPS, capsule formation was very efficient (Fig. 5A). In the supernatant of YPD cultures, EV-like particles were distributed in the typical 50 to 300 nm diameter range. Particles larger than 300 nm were not observed. EV-like particles from RPMI had discrete peaks at 300 to 350 nm and 400 to 500 nm. Particles obtained from MOPS were distributed into three major ranges: 100 to 250, 250 to 300, and 400 to 600 nm, with an unequivocal detection of larger particles. These results supported the hypothesis that capsule formation involves the participation of larger, GXM-containing EVs, and indicates that the type and amount of EVs is directly influenced by the culture conditions. Under the conditions used in our model, the highest detection of EVs occurred in YPD (approximately 5 EVs per cell), followed by Sabouraud (roughly 2 EVs per cell), MOPS (nearly 1 EV per cell), and RPMI (less than 0.5 EVs per cell) (Fig. 5B).

**FIG 6:**
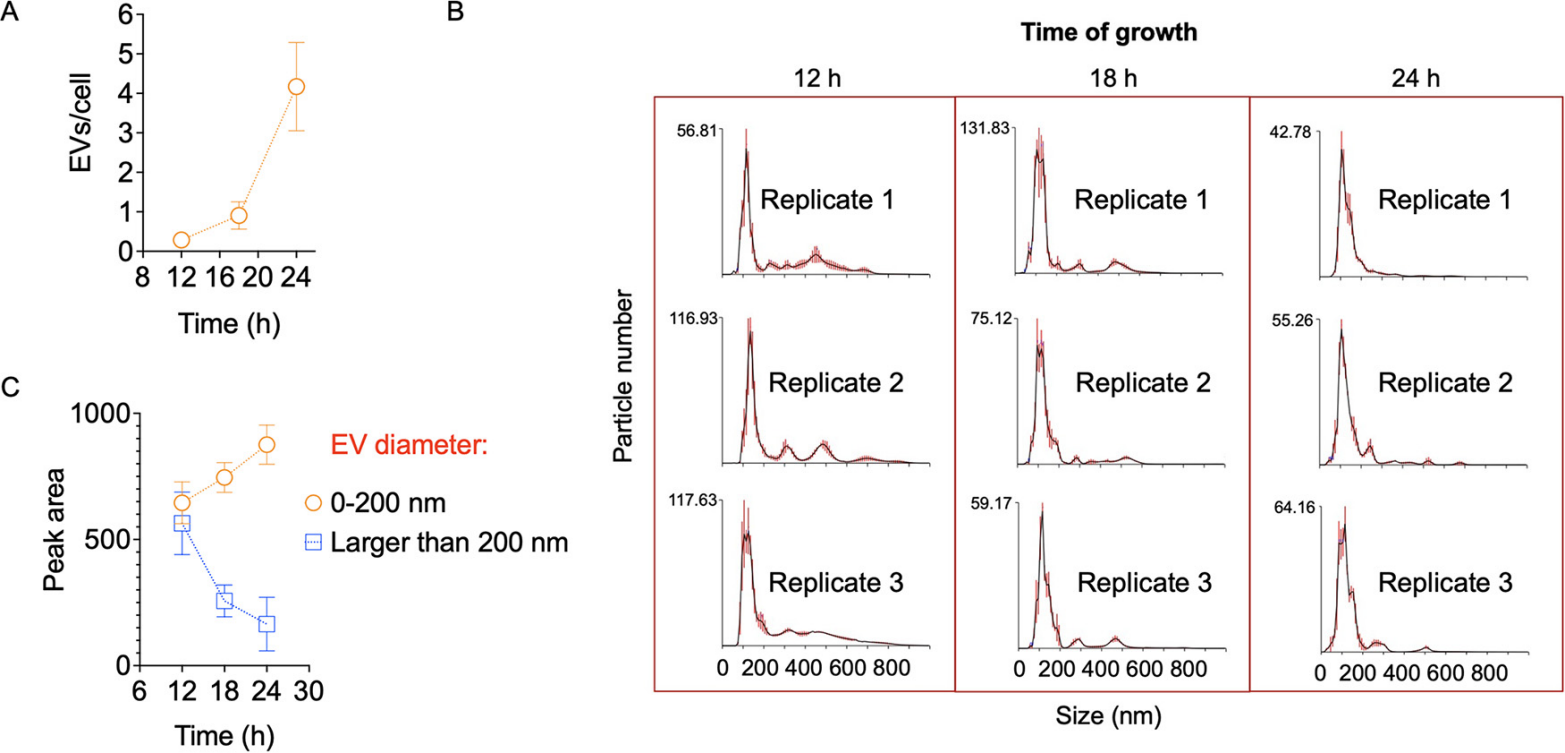
Kinetics of EV formation in solid YPD. (A) Detection of EVs at 12, 18, and 24 h postinoculation. The peak of EV detection (normalized to the cell number) was observed at 24 h of growth. (B) Individual NTA profiles of the samples described in A, revealing that the size of the EVs varied with the time of cultivation. (C) Quantification of smaller (0 to 200 nm) and larger (>200 nm) particles in the samples illustrated in B, using the Image J software. Shorter cultivation times were associated with the increased detection of larger EVs, while the observation of smaller EVs increased in response to the time of growth.

We returned to the solid medium model with an aim to expand the qualitative and quantitative results described in Fig. 3 and Fig. 5. We then initially analyzed the kinetics of EV formation until 24 h of cultivation in solid YPD. Our initial time point was 6 h, but the amount of living cells was not sufficient for EV isolation (data not shown). However, EVs were detectable at 12, 18, and 24 h postinoculation (Fig. 6A). The peak of EV detection (normalized to the cell number) was observed at 24 h of growth (approximately 4 EVs/cell). Unexpectedly, NTA revealed that the size of the EVs also varied with the time of cultivation (Fig. 6B). Based on the NTA profiles, we classified the EVs into smaller (0 to 200 nm) and larger (>200 nm) particles for further quantification of each population. Shorter cultivation times were apparently related to the increased detection of larger EVs (Fig. 6C). The observation of this specific population decreased with time, while the observation of smaller EVs increased in response to the time of growth. These results suggested an unexpected relationship of not only the amount of EV formation with time, but also with the type of vesicles.

**FIG 7:**
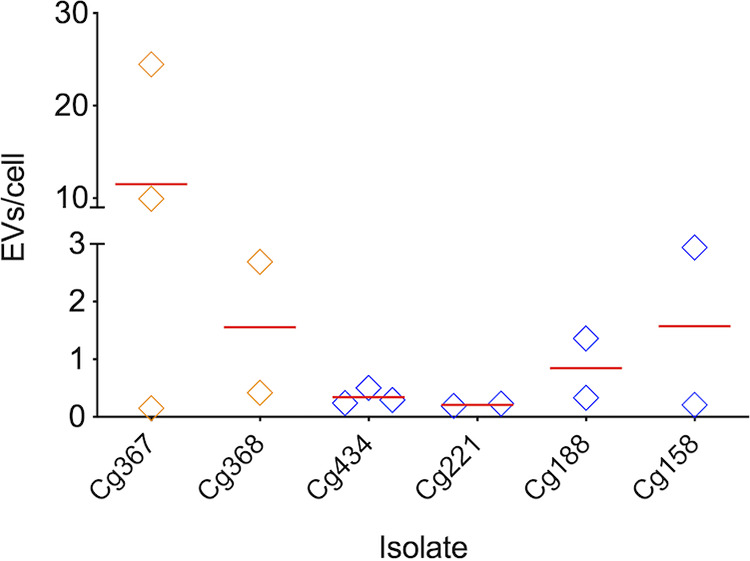
Quantification of EVs produced by isolates of C. gattii (orange diamonds) or *C. deuterogattii* (blue diamonds) in solid YPD. EV detection varied from less than 1 to more than 20 particles per cell, with average values represented by the red dashes. Data in this figure originated from duplicates or triplicates, with individual values represented by the diamonds.

Our results of EV observation in both solid and liquid media revealed a range of detection corresponding to 0.5 to 4 EVs per cell, approximately. To make sure this number was reflecting a property of the Cryptococcus genus rather than being a particularity of the R265 *C. deuterogattii* isolate, we extended our analysis of EV quantification to 2 isolates of C. gattii and 4 strains of *C. deuterogattii*. EV detection varied from less than one to more than 20 vesicles per cell (Fig. 7). These results, once again, confirmed the diversity in the formation of EVs in Cryptococcus.

### EVs were detected in Titan cell cultures obtained in a solid medium.

**FIG 8:**
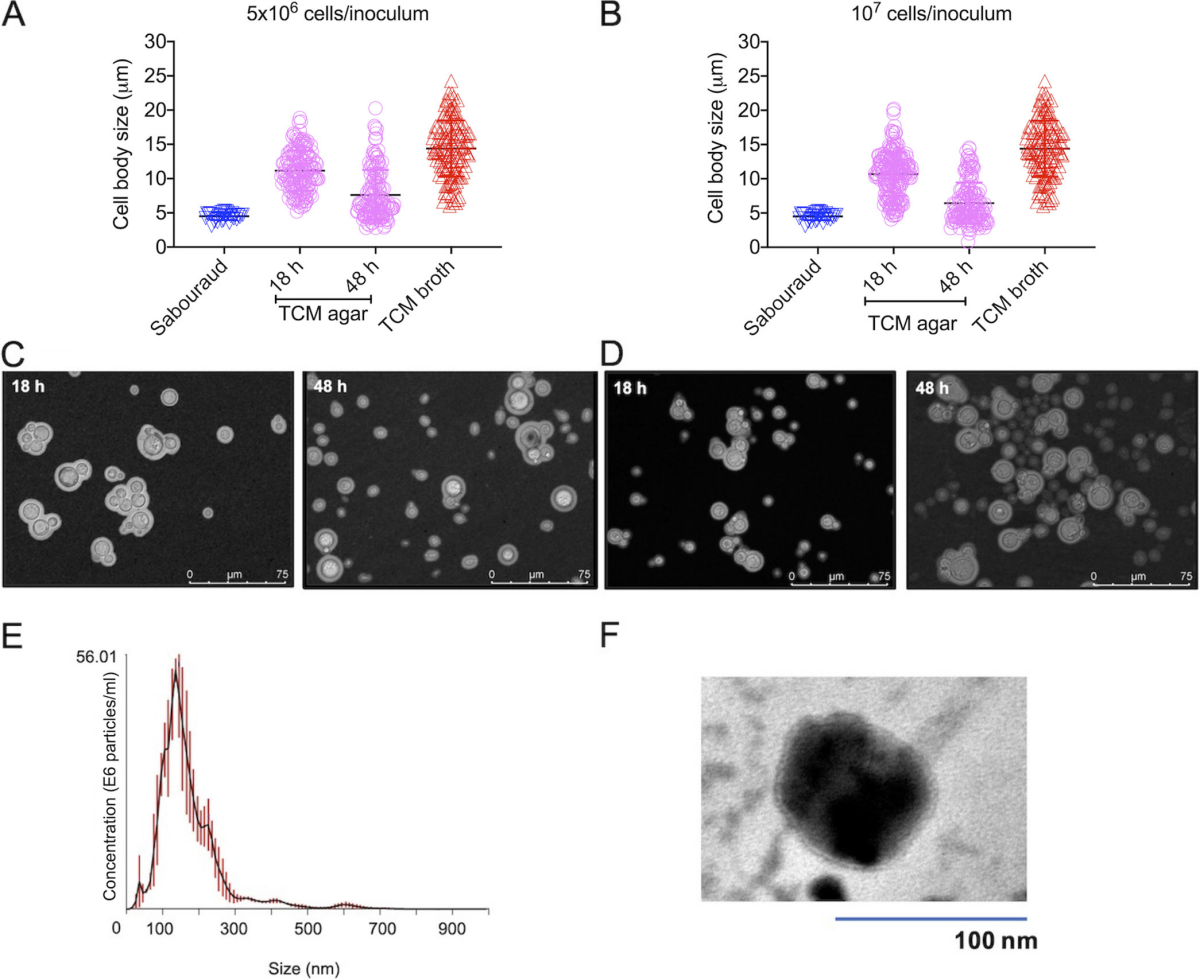
Analysis of Titan cells cultivated in solid medium. C. neoformans (H99) was inoculated in solid Sabouraud, TCM agar, or TCM broth at 5 × 10^6^ (A and C) or 10^7^ cells per plate (solid medium) or tube (TCM broth, 10^4^ cells/ml in 96-well plates) (B and D). The cells were incubated for 18 or 48 h for measurement of cell body size, as illustrated in panels A and B. The visual analysis of each condition is shown in panels C and D. (E and F) Incubation of an inoculum of 10^7^ cells for 18 h in TCM agar was selected as the condition for EV isolation and analysis by NTA (E) and TEM (F). These results illustrate one out of three experiments producing similar results.

We selected the strain H99 of C. neoformans for the Titan cell assays, based on the previously reported efficacy of this strain for differentiating into large cells *in vitro* (30, 38, 39). To use the solid medium protocol of EV isolation in cultures of Titan cells, we first asked whether C. neoformans could differentiate into this morphological stage in solid cultures. Therefore, we used a solid formulation of the so-called Titan cell medium (TCM) (30) and searched for the best conditions to result in the formation of larger cells. We tested two distinct cell densities to inoculate the solid medium plates (5 × 10^6^ or 10^7^ cells per plate), and incubated the cultures for two different periods (18 or 48 h). The cell body size was measured in all systems and compared to the results obtained under the previously optimized conditions for Titan cell formation in liquid medium (30). Incubation of the cells in solid Sabouraud consisted of a negative control of Titan cell formation. In all sets of experiments, Titan cell formation was more efficient under the conditions using liquid TCM (Fig. 8A to D). However, Titan cells were also efficiently formed in solid cultures, as evidenced from microscopic observation and measurement of cell body sizes. The results using 5 × 10^6^ or 10^7^ cells per plate were similar, but the 18 h of incubation time resulted in a more efficient Titan cell formation than the 48 h period. We then selected 10^7^ cells per plate incubated for 18 h as the condition used for EV isolation. The EV fractions were submitted to NTA, which revealed the typical pattern of EV diameter distribution (Fig. 8E). Since EVs from cultures of Titan cells have never been observed, we also analyzed these fractions by transmission electron microscopy (TEM) after negative staining of the vesicle samples. TEM analysis revealed the presence of double-layered membranes that were morphologically compatible with fungal EVs (Fig. 8F). These results indicate that Titan cells can be formed in solid medium, which apparently is a functional matrix for the isolation of EVs.

## DISCUSSION

The knowledge on fungal EVs has expanded substantially since their first description in 2007 (1). However, it is consensual in the field that several fundamental questions still have no answer (28, 35). For instance, it is clear that fungal EVs have multiple sites of biogenesis (3), including endosome-like structures and the plasma membrane (1, 5), but the molecular regulators of EV formation remain unknown. In this sense, several candidates have been suggested (9), but shutting down EV production has not been successful. This observation agrees with the supposition that EVs have multiple sites of origin in fungi but reveals a major hurdle for the functional studies of fungal vesicles, since knowing the molecular regulators of EV formation is essential for both genetic and pharmacological approaches to inhibit EV formation.

The separation of EV samples into subpopulations with similar properties for further compositional analysis will likely provide insights into the genetic and/or pharmacological control of EV production, since EVs of different origins carry molecular markers of their sites of biogenesis (31). In this sense, our current study reveals that EVs of distinct properties are differentially separated on a density gradient. We have tried to surpass this issue for more than a decade, but a common issue with EV samples obtained from liquid media was the difficulty in preparing concentrated samples that could be separated and recovered in detectable amounts in the density gradient (32). This is especially challenging in the Cryptococcus model, since our own empirical experience and data from colleagues in the field indicate that other yeast cells, including *Candida* and *Saccharomyces*, produce EVs in much higher amounts. To illustrate the applicability of separation models in biogenesis studies, our density gradient model suggested an association between larger EVs and the presence of GXM. This supposition was supported by the direct detection of EVs in culture supernatants. In these assays, EVs were larger and less numerous after induction of capsular enlargement, which agrees with a previous suggestion that EVs are a source of GXM for capsule growth (1). However, additional features influence the incorporation of GXM into the surface of acapsular cells. In our gradient separation, fractions with low GXM concentration (fractions 2 to 4) functioned as effective sources for polysaccharide incorporation into the surface of acapsular cells. In a recent study, we observed that the membrane structure of EVs affect the effectiveness of GXM incorporation by acapsular cells (2). Therefore, if the vesicles separated in fractions 2 to 4 differ in membrane composition from those detected in other fractions, the effectiveness of GXM incorporation by acapsular cells could be directly affected.

Microvesicles are larger EVs formed by plasma membrane budding and extracellular release, which have been observed in cryptococcal cells (1, 3). In this context, our results could indicate that polysaccharide-containing vesicles correspond to microvesicles instead of exosomes (40). However, GXM is highly hydrophilic, and the assimilation of water into the EVs due to the presence of the polysaccharide would directly influence the vesicle dimensions. In this case, the presence of GXM itself could be the reason explaining the detection of enlarged vesicles, and not necessarily their mechanisms of biogenesis. Exosomes, the most well-studied eukaryotic EVs, derive from the endocytic pathway (41). Microvesicles are usually larger in size than exosomes (40) and this property might be reflected in our analysis. In this study, larger EVs were produced at early stages of fungal growth, while smaller EVs were more abundant at late stages. These results suggest a differential regulation of EVs of different cellular origins in Cryptococcus.

Another methodological improvement proposed in this study was the direct detection of EV particles in culture supernatants. Although the rapid detection of EV in crude samples is certainly not conducive to fine vesicle characterization, it might be useful for rapid and large screenings followed by more refined methodologies. Direct EV detection, therefore, could be useful for the screening of drug libraries and/or mutant collections, aiming at identifying pharmacological inhibitors and genetic regulators of EV production, respectively.

Due to the easier EV isolation and better yields, the solid medium protocol was also useful for the analysis of an isolate collection. In our study, we initially tried to prove that EV analysis could be scaled up from a few isolates to at least a dozen. Using the previously described protocols, EV detection in cryptococcal samples would have required 0.5 to 1 liter of supernatants for each isolate, which would require processing 7.5 to 15 liters of cultures for each replicate of the 15 isolates tested in our study. With the solid medium protocol, this was enormously facilitated, which allowed us to prepare triplicates of each of the 15 isolates in a matter of weeks. NTA of these samples led us to the unexpected finding that cryptococcal EVs have variable properties among different isolates. The impact of these results on immunological studies is still unpredictable, but the concept that different strains produce different EVs was proved. Most of the studies using fungal EVs to stimulate host cells were based on a few standard isolates. If one considers that different subpopulations of EVs have different sites of biogenesis (40), they will have different compositions, as extensively described in the eukaryotic literature (31). Different compositions will likely result in distinct immunological functions. Fungal EVs were proposed as vaccine candidates (25), but in certain models, they favor the disease progress (16). An extended analysis of EVs produced by multiple isolates can reveal more promising vaccine candidates, but it can also result in the characterization of EVs with opposing functions. In this sense, our results suggest that is highly recommendable that studies on the functions of EVs include multiple isolates.

Titan cell formation is a unique event related to the pathogenesis of Cryptococcus (42). The cellular and molecular regulators of Titan cell formation are still superficially known, as well as the physiological and metabolic events regularly occurring in these cells. There is no evidence in the literature that Titan cells of Cryptococcus produce EVs. Our initial attempts to detect EVs produced by Titan cells in liquid medium failed (H. C. de Oliveira, unpublished observation), and it remained unknown whether Titan cells were unable to produce EVs or if we could not detect them due to low yields in the isolation methods. We applied the solid medium protocol in the Titan cell model and obtained particles that corresponded to EVs. Noticeably, we did not obtain 100% of Titan cells in the solid medium, so the possibility that the EVs that we detected were produced by regular cells could not be ruled out. However, our preliminary results on the proteomic characterization of Titan cell EVs revealed marked differences in the composition of vesicles obtained from enlarged and regular cells in solid media (data not shown). Therefore, we believe Titan cells can be added to the list of fungal cells producing EVs, which currently include yeast cells, spores, mycelia, and biofilms.

The understanding of how fungal EVs are formed and what they do in the extracellular space is still limited, and the number of questions behind these phenomena is substantial. Our aim in the current study was to propose experimental approaches to address several open questions in the field of fungal EVs, in addition to the consideration of still unexplored parameters during the analysis of the functions of these structures. Our methods and related results can contribute to the design of future experimental models aiming at exploring the mechanisms of biogenesis and biological functions of fungal EVs.

## MATERIALS AND METHODS

### Fungal strains.

The cryptococcal strains used in this study included the standard strains H99 of C. neoformans and R265 of *C. deuterogattii*, in addition to human and environmental isolates that were recently characterized at the phenotypic and genomic levels (43). The isolates included C. neoformans (Cn271, Cn216, Cn186, Cn161, Cn115, and Cg366), *C. deuterogattii* (Cg221, Cg461, Cg434, Cg158, and Cg188), and C. gattii (Cg198, Cg456, Cg367, and Cg368) strains. The conditions used for the cultivation of each strain will be described in the following sections.

### EV isolation from solid medium.

We used our recently described protocol for EV isolation from solid medium (2). Briefly, one colony of each isolate cultivated in solid Sabouraud’s medium was inoculated in yeast extract-peptone-dextrose (YPD, 5 ml) medium and cultivated for 24 h (30°C, with shaking). The cells were counted and adjusted to 3.5 × 10^7^cells/ml in YPD. These cells (300 μl) were inoculated onto YPD agar plates and incubated for 24 h at 30°C. For each isolate, the cells were gently recovered from each of the three plates with an inoculation loop and suspended in 30 ml of phosphate-buffered saline (PBS). The suspensions were centrifuged at 5,000 × *g* for 15 min at 4°C for removal of the cells. To remove debris, the supernatants were collected and centrifuged again at 15,000 × *g* for 15 min at 4°C. The resulting supernatants were filtered through 0.45-μm pore syringe filters and centrifuged at 100,000 × *g* for 1 h at 4°C for pelleting down the EVs. For the gradient separation, the EV samples were filtered through 0.8 μm membranes, to avoid the loss of larger EVs. The protocols used for the kinetics of EV production and fungal growth rates were similar to those described here, except that the cultures were maintained in YPD-agar plates for 12, 18, and 24 h of growth.

### Nanoparticle tracking analysis of EVs.

The method used for monitoring the presence of EVs in our samples was the nanoparticle tracking analysis (NTA). This analysis was performed on an LM10 nanoparticle analysis system, coupled with a 488-nm laser and equipped with an _S_CMOS camera and a syringe pump (Malvern Panalytical, Malvern, United Kingdom). All samples were measured according to the conditions previously described by our group (2). The data were acquired and analyzed using the NTA 3.0 software (Malvern Panalytical). Particle numbers obtained by NTA were normalized to the number of cells in the cultures for determination of the EV/cell ratios. NTA histograms were also used for the quantification of EV peaks in different samples using the Image J software (https://imagej.nih.gov/ij/). In the gradient samples, diameter ranges corresponded to 300 to 400 nm, 400 to 600 nm, and 600 to 900 nm. In the kinetics studies, EV diameters were simply separated into 0 to 200 nm and larger than 200 nm. In both assays, these regions of the histograms were manually colored using the Image J paintbrush tool, and their areas were automatically measured using the wand/tracing tool.

### Gradient centrifugation of EVs.

Discontinuous iodixanol gradients were prepared according to Tauro and colleagues (44). Briefly, solutions of iodixanol 40% (wt/vol), 20% (wt/vol), 10% (wt/vol), and 5% (wt/vol) were prepared by diluting OptiPrep in 0.25M sucrose/10 mM Tris, pH 7.5. The first layer of the gradient added to the bottom of the ultracentrifuge tube was formed by 3 ml of the 40% iodixanol solution. Additional layers were carefully poured and consisted of 3 ml of the 20% iodixanol solution, 3 ml of the 10% iodixanol solution, and 2.5 ml of the 5% iodixanol solution. EVs obtained from 5 YPD agar plates were suspended in 0.5 ml of PBS and added on the top of the gradient. The tubes were ultracentrifuged (100,000 × *g*) for 18 h at 4°C. Fractions of 1 ml were collected from the top to the bottom of the gradient and placed in separate tubes. The fractions were washed with 10 ml PBS by ultracentrifugation at 100,000 × *g* for 1 h at 4°C. Individual fractions were suspended in 50 μl PBS and submitted to NTA. For GXM quantification, aliquots of each fraction containing the same number of particles (7 × 10^8^, as estimated by NTA) were vacuum dried and GXM was precipitated through the addition of 100 μl of a mixture of chloroform and methanol (1:2 [vol/vol]). The dried precipitates were delipidated by similar rounds of precipitation using other mixtures of chloroform and methanol (2:1 and 9:1 at 100 μl each), and finally dissolved in 50 μl of PBS for GXM quantification by enzyme-linked immunosorbent assay (ELISA) (45). For analysis of the presence of RNA, the EVs were diluted in 700 μl QIAzol lysis buffer from the miRNeasy isolation kit (Qiagen). The RNA extraction was performed according to manufacturer’s instructions. For RNA quantification, we used an Agilent 2100 Bioanalyzer; RNA 6000 pico kits (Agilent Technologies).

### GXM recovery from EV samples by acapsular cells.

Acapsular C. neoformans cells (*cap67*Δ strain, 5 × 10^6^ cells) were suspended in 150 μl of PBS containing 7.8 × 10^8^ EV gradient particles, as determined by NTA. The systems were incubated at room temperature for 24 h. After incubation, the cells were extensively washed with PBS and processed for immunofluorescence as previously described by our group (2), using a monoclonal antibody to GXM (MAb 18B7) kindly donated by Arturo Casadevall (Johns Hopkins University, Baltimore). The cells were analyzed with a fluorescence-activated cell sorter (FACS) Canto II flow cytometer. Data were processed with the FACSDiva software, version 6.1.3.

### Capsule induction and direct EV detection.

The media used in this assay consisted of YPD, Sabouraud, RPMI, and 10% Sabouraud in 50 mM MOPS, pH 7.3. *C. deuterogattii* (R265) cells were grown for 24 h in YPD at 30°C with shaking. The cells were washed in and suspended at 2.5 × 10^8^/ml in each specific medium. These suspensions were placed into the wells of a 24-well plate (1.2 ml per well). The plates were incubated for 24 h at 37°C under a 5% CO_2_ atmosphere. The content of each well was transferred to 1.5-ml plastic tubes, which were centrifuged at 10,000 × *g* for 2 min. The supernatants were filtered through 0.45-μm membranes and directly submitted to NTA (1:2 dilution). The cells were counterstained with India ink for microscopic observation.

### Formation of Titan cells in solid medium.

For analysis of Titan cells, we prepared a solid form of the induction medium previously characterized for enlargement of cryptococcal cells, the so called Titan cell medium (TCM; 5% Sabouraud, and 5% inactivated fetal calf serum diluted in 50 mM MOPS, pH 7.3, supplemented with 15 μM sodium azide) (30). Agar (2%) was added to 50 mM MOPS (pH 7.3) for autoclave sterilization. This mixture was then supplemented with 5% liquid Sabouraud, 5% inactivated fetal calf serum, and 15 μM sodium azide. The serum was previously depleted of EVs by 100,000 × *g* ultracentrifugation for 16 h followed by filtration through 0.22-μm membranes. The medium was distributed onto petri dishes of TCM agar. To induce the formation of Titan cells, C. neoformans H99 was grown overnight in liquid Sabouraud supplemented with 15 μM sodium azide at 30°C with shaking (150 rpm). The cells were washed with PBS and 2.5 × 10^7^ and 5 × 10^7^ cells/ml suspensions were prepared in PBS. These suspensions (200 μl) were inoculated onto TCM agar plates, leading to a final density of 5 × 10^6^ or 10^7^ cells/plate. The plates were incubated for 18 or 48 h at 37°C with 5% CO_2_. For measurement of cell body sizes, the cells were scraped from the TCM agar plates, suspended in PBS, and mixed with India ink for further observation under a DMi8 microscope (Leica). Images were recorded with the LasAF software, and processed with ImageJ. C. neoformans H99 cells were considered Titan cells when the cell body size was larger than 10 μm. As a control, we induced Titan cell formation in TCM as previously described (30).

### EV isolation from TCM agar.

C. neoformans H99 was cultivated on TCM agar plates (10^7^ cells/plate, 37°C with 5% CO_2_ for 18 h). The cells were collected from the plates, suspended in 30 ml of PBS, and the EV isolation followed the protocol described above, with the inclusion of one additional ultracentrifugation step (100.000 × *g*; 1 h) to pellet EVs. After the second ultracentrifugation, EVs were suspended in 100 μl of PBS and analyzed by NTA. Alternatively, the EVs were processed for transmission electron microscopy (TEM). The samples were transferred to carbon- and Formvar-coated grids and negatively stained with 1 % uranyl acetate for 10 min. The grids were then blotted dry before immediately being observed in a JEOL 1400Plus transmission electron microscope at 90 kV.
